# Microscopic Description of the Ferroism in Lead-Free AlFeO_3_

**DOI:** 10.1038/s41598-018-24880-4

**Published:** 2018-04-23

**Authors:** Guilherme M. Santos, Igor B. Catellani, Ivair A. Santos, Ruyan Guo, Amar S. Bhalla, José Eduardo Padilha, Luiz F. Cótica

**Affiliations:** 1 Department of Physics, State University of Maringá, Maringá, PR 87020-900 Brazil; 2 Department of Electrical and Computer Engineering, University of Texas at San Antonio, San Antonio, TX 78249 USA; 3 Campus Avançado de Jandaia do Sul, Federal University of Paraná, Jandaia do Sul, PR 86900-000 Brazil

## Abstract

The microscopic origin of the ferroic and multiferroic properties of AlFeO_3_ have been carefully investigated. The maximum entropy method was applied to X-ray diffraction data and *ab initio* density functional theory calculations in order to obtain the electron density distributions and electric polarization. The study of chemical bonds shows that the bonds between Fe(3d) and O(2p) ions are anisotropic, leading to the configuration of shorter/longer and stronger/weaker bonds. This leads to electric polarization. Density of states calculations showed a magnetic polarization as a result of a weak ferromagnetic ordering. These results unambiguously show that AlFeO_3_ is a multiferroic material and exhibits a magnetoelectric coupling at room temperature, as has already been shown by experiments.

## Introduction

Combining different physical properties into one achievable material is a very promising approach for the creation of multifunctional materials. From the physical point of view, multiferroics represent an extremely interesting class of such materials. They can exhibit simultaneous coupling between at least two of the primary ferroic order parameters: ferromagnetism, ferroelectricity and ferroelasticity^1^. Exploration of these multifunctional features of multiferroics, in particular the magnetoelectric effect, has been gaining interest due to their intriguing physics and potential application in memories, sensors, and transducers^2^.

Most magnetoelectric materials, such as BiFeO_3_ and Pb(Fe1/2Nb1/2)O_3_, include Pb and Bi^2,3^. However, due to the toxicity of lead and bismuth, it is necessary to search for materials that exhibit similar properties but are less harmful to the environment. Among many possible candidates, AlFeO_3_ (AFO) emerged as an alternative lead-free multiferroic material. This material exhibits piezoelectricity and weak ferrimagnetic behavior at low temperatures^4–9^. AFO has an orthorhombic crystal symmetry (*Pna*2_1_ space group), and the crystal structure has four inequivalent iron and aluminum ionic sites, similar to GaFeO_3_, and *ε*-Fe_2_O_3_^9,10^. Three of these, named Fe1, Fe2 and Al2, are in an octahedral environment, and the other one, the Al1 site, has a tetrahedral environment. In addition, this material exhibits a disordered structure due to the mixture between Fe^3+^ and Al^3+^ along the four cationic sites, showing different ratios of iron and aluminum in each site. Regarding the ferroic properties at room temperature, AFO was found to exhibit ferroelectric relaxor behavior and magnetic spin-glass behavior with a weak ferromagnetic ordering^5^. Due to the coexistence of these particular magnetic and ferroelectric states at the same temperature, AFO can be considered as a multiferroic material with a non-linear magnetoelectric (NLME) response at room temperature^6^.

At room temperature, the linear magnetoelectric behavior is a weak phenomenon for AFO. However, as addressed by Blinc *et al*.^11,12^, a NLME behavior occurs for a material with the combination of magnetic nanoregions (magnetic spin-glass) and ferroelectric nanoregions (relaxor). Thus, while the magnetoelectric coupling occurs at the nanoscale, it promotes macroscopic responses, as reported in a previous work^6^. In fact, for magnetically frustrated systems, NLME effects have been proposed in materials where the magnetic disordered (magnetic spin-glass) and relaxor ferroelectric states coexist. In this scenario, the AFO, at room temperature, is a ferroelectric relaxor (T_*f*_ = 230 K) and a magnetic spin-glass (T_*m*_ = 240 K) compound, emerging as a single-phase material that can exhibit magnetoelectricity at room temperature. In addition, this nonlinear effect can be studied using the fact that the polarization of a nanoregion (presenting locally ferroelectrically ordered structures) can be nonlinearly increased by applying an external magnetic field (H_*ext*_). Actually, such systems exhibit strong fourth-order magnetoelectric coupling in the free energy of the E^2^H^2^ type^11,12^. By applying external magnetic fields (H_*bias*_ and h_*ac*_), the polarization of the polar nanoregions in AFO increases, leading to the NLME behavior as reported in previous work^6^.

However, the understanding of the magnetoelectric phenomena in AFO remains an open issue. Investigations from the fundamental point view are desirable, such as examination of the orbital hybridization, which could clarify the covalent behavior of the bonds in the unit cell that has been identified as the driving force in the emergence of electric polarization^13^. To gain more insights into the electronic properties of AFO, this fundamental issue can be achieved using *ab initio* calculations based on density functional theory (DFT)^14,15^, which provides accurate results for the atomic structure and stability, electrical polarization, electron density maps, density of states and band structure.

Similarly, the maximum entropy method (MEM) is a powerful approach for obtaining electron density information and bonding structure experimentally. MEM is a well-established high-resolution technique commonly used for a precise reconstruction of the electron density maps from X-ray diffraction data^16,17^. It is also more accurate for the reconstruction of electron densities than the use of inverse Fourier transforms because it overcomes the issue of information loss (experimental noise) by maximizing the information entropy^16^.

In this work, we discussed the origin of ferroic and multiferroic properties in the lead-free AFO material by studying its structural and electronic properties. The chemical bonds and the electronic structure were investigated using the electron density, density of states and band structure and electrical polarization calculations. Indeed, we show that the displacement of the cations around the octahedral and tetrahedral environments can be an origin of the ferroelectric properties and, due to the mixture between the aluminum and iron ions at the cationic sites, weak ferromagnetism arises as the magnetic ordering in the AFO material.

## Results

**Figure 1:**
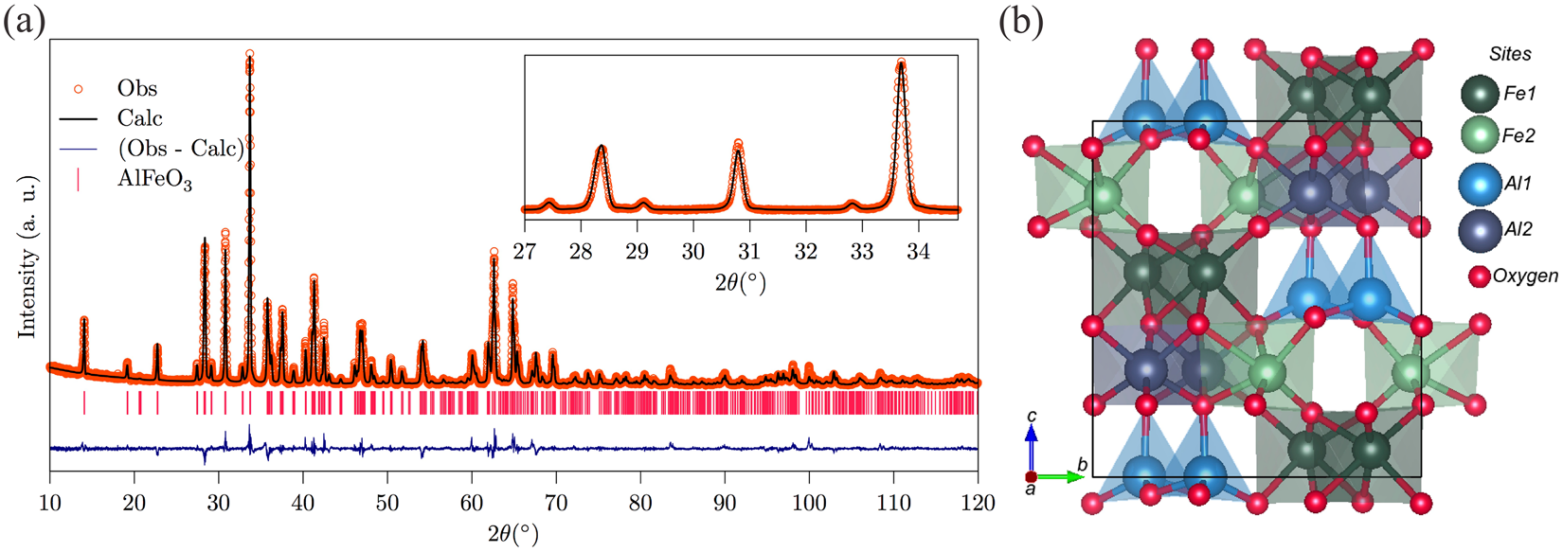
(**a**) Rietveld-refined XRPD pattern of AlFeO_3_. (**b**) AlFeO_3_ crystal structure highlighting the four different cationic sites.

Figure 1(a) depicts the XRPD pattern obtained for the AFO sample, and in (b) we present the structure obtained from the refinement analysis. Rietveld analysis confirmed that the AFO material crystallizes in an orthorhombic structure with the *Pna*2_1_ space group containing eight unit formulas with 40 atoms in the unit cell, in the same way as for GaFeO_3_ and *ε*-Fe_2_O_3_^9,10^. Inspection of Fig. 1(a) shows a good match between the Rietveld fit and the data obtained from X-ray powder diffraction (*χ*^2^ = 2.65). The refinement results are summarized in Table 1 and are in good agreement with the results obtained by Bouree *et al*.^4^ and by Cotica *et al*.^5,6,18^.

**Table 1:** Lattice parameters, atomic positions and site occupancy of AlFeO_3_ at room temperature as obtained in the Rietveld refinement.

Crystallographic data of AlFeO_3_						
Symmetry	Orthorhombic	Atomic Position/Site Occupancy				
Space Group	*Pna*2_1_	Sites	*x/a*	*y/b*	*z/c*	Occ
*a*	4.9902 (9) Å	Fe1/Al	0.1842(8)	0.1524(5)	0.5757(1)	0.7721(9)/0.2179(9)
*b*	8.5626 (6) Å	Fe2/Al	0.6680(6)	0.0314(3)	0.7907(1)	0.7036(7)/0.2864(7)
*c*	9.2542 (7) Å	Al1/Fe	0.1771(1)	0.1565(1)	−0.0008(1)	0.8325(4)/0.1475(4)
Volume	395.43 (13) Å^3^	Al2/Fe	0.8115(9)	0.1631(5)	0.2979(1)	0.9556(7)/0.0244(7)
Density	4.135 g/m^3^	O1	1.0073(3)	0.3223(2)	0.4266(2)	0.7587(1)
		O2	0.4870(2)	0.5022(2)	0.4190(2)	0.8446(2)
Reliability Factors		O3	0.6566(3)	1.0060(1)	0.2009(2)	1.0
R_*wp*_	11.45%	O4	0.1819(3)	0.1608(2)	0.2003(2)	0.9120(2)
R_*e*_	7.03%	O5	0.8662(3)	0.1667(1)	0.6798(2)	1.0
*χ* ^2^	2.65	O6	0.5167(1)	0.1762(1)	0.9500(5)	0.9800(1)

**Figure 2:**
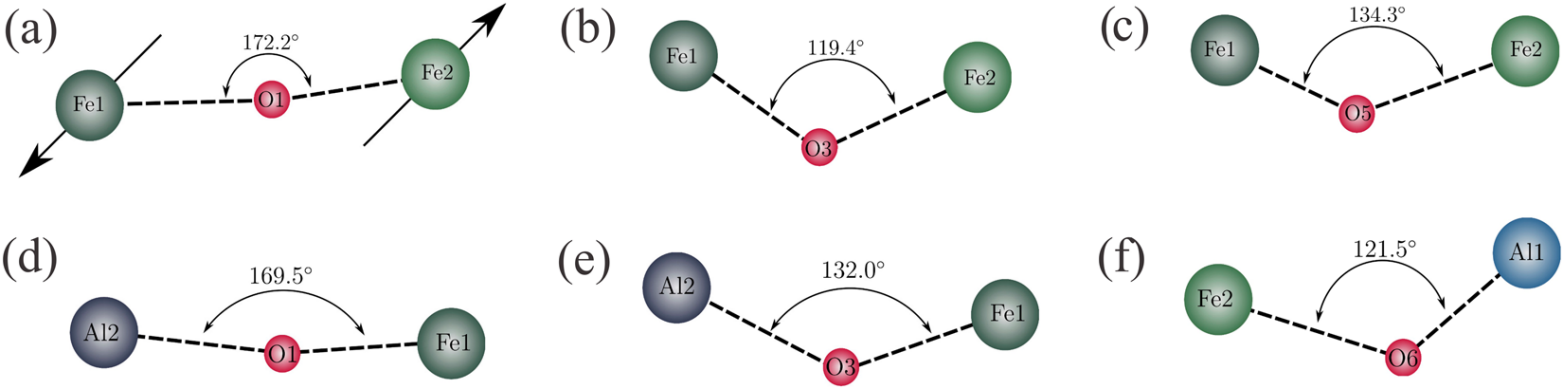
Schematic diagram showing the angle between the different cation sites bonds across the oxygen sites. (**a**) Magnetic moments in opposite directions in Fe1 and Fe2 sites due to the “180° cation-anion-cation” superexchange antiferromagnetic interaction and the Fe1-O1-Fe2 angle. The angles related to the (**b**) Fe1-O3-Fe2, (**c**) Fe1-O5-Fe2, (**d**) Al2-O1-Fe1, (**e**) Al2-O3-Fe1 and (**f**) Fe2-O6-Al1 bonds are shown.

Regarding the magnetic properties, due to the “180° cation-anion-cation” superexchange antiferromagnetic interaction, AFO exhibits an antiferromagnetic structure with the magnetic moments in the opposite directions^4,10^, as depicted in Fig. 2(a). Additionally, we notice that the angle between the Fe1 and Fe2 sites is close to 180°, especially when O1 is located between the anion site. However, the AFO is in a ferrimagnetic state as a result of the different magnitudes for the magnetic moment at each ion site. This feature is due to the disordered structure of AFO, where the Fe^+3^ ions can be located at both Al1 and Al2 sites, and the Al^+3^ ions can be found at both Fe1 and Fe2 sites. In this way, the superexchange interaction occurs even at the Al1 and Al2 sites. This is because some Fe^+3^ ions are present at these sites, as shown in Fig. 2(d–f).

**Figure 3:**
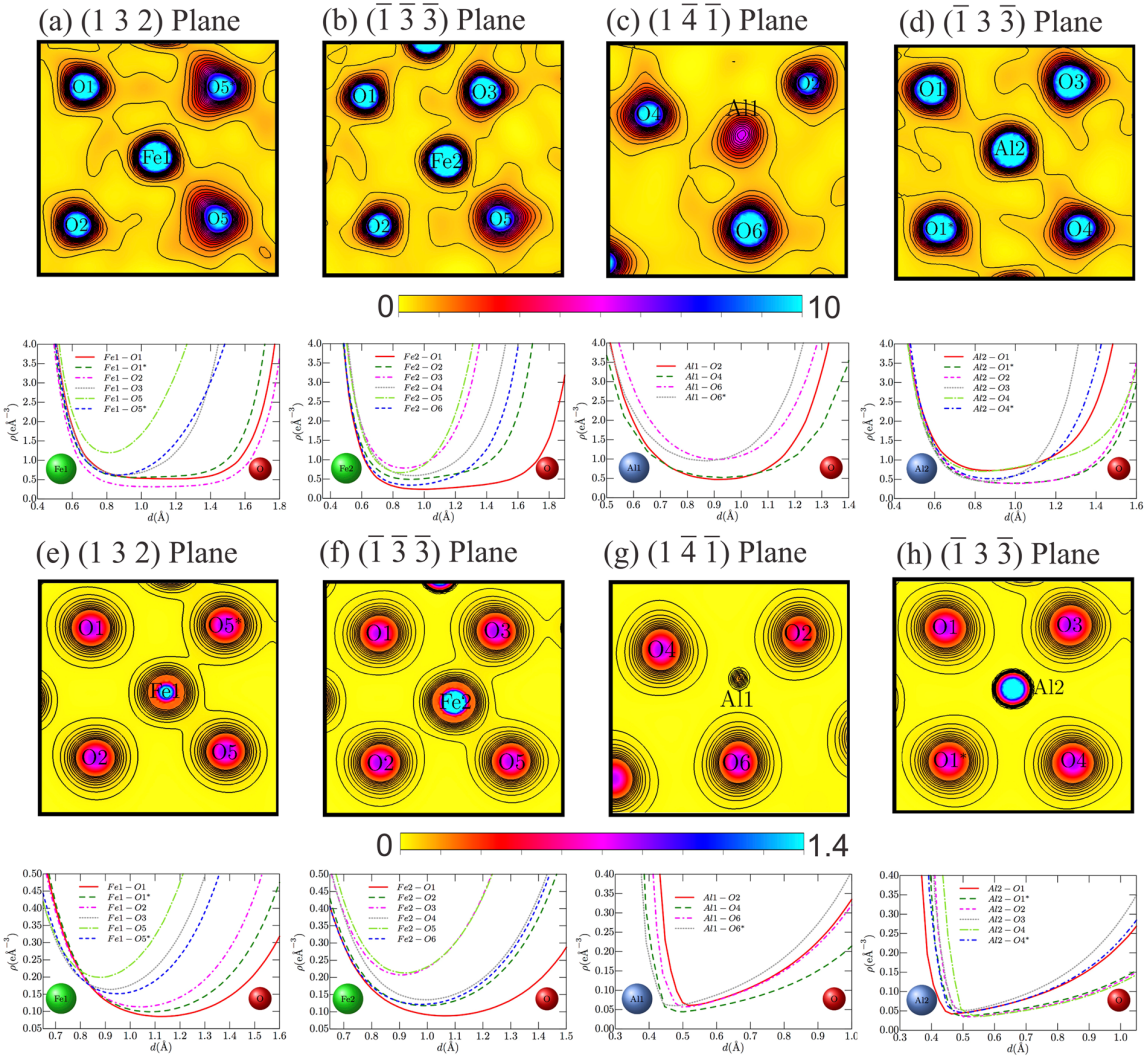
(**a–d**) 2D (top) and 1D (bottom) MEM electron density distributions for AlFeO_3_ obtained from X-ray diffraction measurements at 300 K for the four different cationic sites. (**d–h**) 2D (top) and 1D (bottom) electron density distributions from DFT calculations for the four different cationic sites.

To study the characteristics of the chemical bonds and their influence on the electronic properties of the AFO compound, the electron density maps were obtained by both MEM and DFT calculations. The behavior of the cationic electron density in relation to the anions in the proximity to the cation was analyzed for each cationic site. The 2D MEM maps (top) and the one-dimensional electron density between the oxygen ions and the cationic sites (down) are shown in Fig. 3(a–d). These are drawn in the 0 to 10 *e*/Å^3^ range on the (132) plane for the Fe1 site (Fe1(3*d*)/Al(2*p*)-O(2*p*) bonds), on the ($\bar{1}\bar{3}\bar{3}$) plane for the Fe2 site (Fe2(3*d*)/Al(2*p*)-O(2*p*) bonds), on the ($1\bar{4}\bar{1}$) plane for the Al1 site (Al1(2*p*)/Fe(3*d*)-O(2*p*) bonds) and on the ($\bar{1}3\bar{3}$) plane for the Al2 site (Al2(2*p*)/Fe(3*d*)-O(2*p*) bonds), respectively. All maps were drawn in steps of 0.65 *e*/Å^3^. The 2D DFT electron density maps (top) and the one-dimensional electron density between oxygen ions and the cationic sites (down) are depicted in Fig. 3(e–h) and are drawn in the 0 to 1.4 *e*/Å^3^ range using the same plane of each site already mentioned for the MEM calculations presented in Fig. 3(a–d). In the case of the DFT calculations, all maps were drawn in 0.12 *e*/Å^3^ steps.

By comparing the results for the Fe1 site obtained from MEM and DFT, it is possible to note some similarities between them, such the higher electron density connecting the Fe1-O5 sites. The same similarities can be observed for the rest of the chemical bonds in the Fe1 sites. These similarities are more evident in the Fe2 site, as depicted in Fig. 3(a) and (b) (obtained from MEM) and Fig. 3(e) and (f) (obtained from DFT), where is possible to recognize that the Fe(3*d*)-O(2*p*) bonds are very similar to each other in both calculations, i.e., the same orbital hybridization and the same covalent bonds can be observed. However, the Al(2*p*)-O(2*p*) bonds pattern changes dramatically when the MEM and DFT maps are compared. In the MEM maps, we can clearly see a covalent bond between the Al1 and Al2 sites and the surrounding oxygen ions. In contrast, the DFT results do not exhibit any covalent bonds, showing only a small charge density around the Al site that is due to the core electrons. This difference between the MEM and the DFT maps could be explained by the disordered character of the AFO composition. In the MEM maps, each cationic site contains the contribution of both Al^+3^ and Fe^+3^ ions in the ratio presented in Table 1. In contrast, the DFT results captures only the Al^+3^ behavior, resulting in the absence of valence electrons in the Al site. This explains the lack of electron density connecting the Al1 and Al2 sites with the oxygen ions in their proximity. In summary, both results complement each other. This conclusion implies that the superexchange interactions can occurs in the Al1 and Al2 cationic sites as a result of the disordered occupancy of these sites.

In addition, using the data presented in the Table 2 and the charge density maps in Fig. 3, it is possible to see that all cations are located away from the centers of their octahedral/tetrahedral environments, leading to the appearance of shorter and longer bond lengths. It is known from previous theoretical DFT studies for other multiferroic materials that the system lower its total energy by changing the exchange interactions, displacing the ions from the centrosymmetric positions and giving rise to the electric polarization^10,19^. In our case, this could be observed when we go from the non-polar centrosymmetric Pnna system to the polar non-centrosymmetric Pna _2_ one.

**Table 2:** Distance in Å and the minimum electron density (MED) in *e*/Å^3^ for the bonds between Fe1, Fe2, Al1 and Al2 sites and the surrounding oxygen ions.

	Distance/MED			
	Fe1	Fe2	Al1	Al2
O1	2.1325(3)/0.522	2.3564(4)/0.236	—	1.9339(3)/**0.725**
O1*	2.1907(3)/0.557	—	—	2.0576(3)/0.399
O2	2.1962(3)/0.317	2.1058(4)/0.492	1.7227(3)/0.466	2.0062(3)/0.391
O3	1.9527(3)/0.623	1.8490(3)/**0.783**	—	1.7920(3)/0.451
O4	—	1.9924(3)/0.589	1.8613(4)/0.521	2.0574(4)/0.704
O4*	—	—	—	1.8736(3)/0.515
O5	1.8607(3)/**1.196**	1.8368(3)/0.664	—	—
O5*	2.0376(3)/0.614	—	—	—
O6	—	2.0690(3)/0.338	1.7030(3)/**0.991**	—
O6*	—	—	1.7629(4)/0.953	—

**Figure 4:**
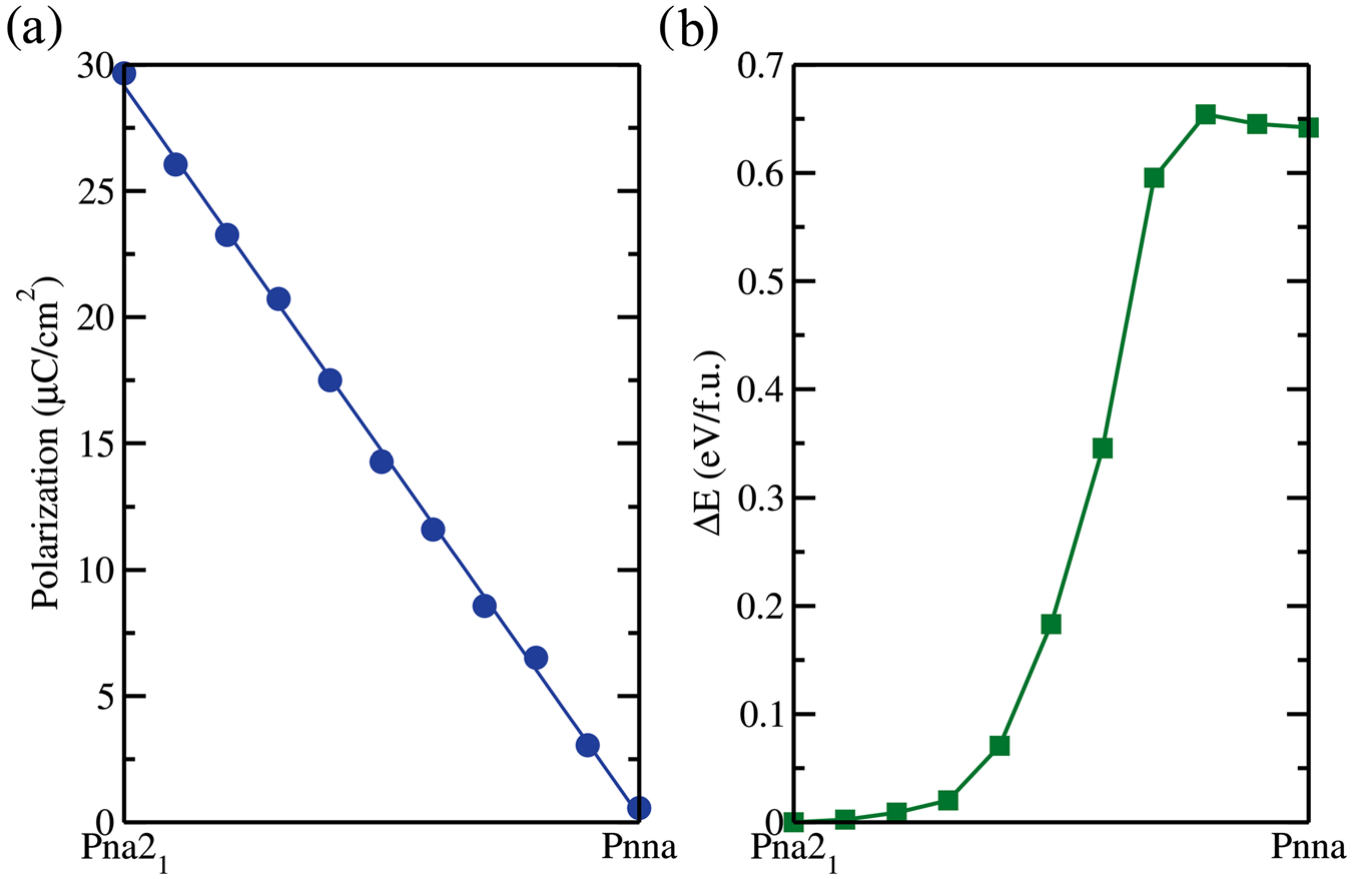
(**a**) Electric polarization of the AlFeO_3_ calculated between the transition path from the polar structure Pna2_1_ to the non-polar structure Pnna. (**b**) Energy difference relative to the Pna2_1_, in the same transition path as in (**a**), from the polar Pna2_1_ symmetry to the non-polar Pnna symmetry.

To calculate the electric polarization, we have followed the work of Stoeffler^9^, that precisely calculate the electric polarization through first principles calculations of the GaFeO_3_ system. The electric polarization of the system was determined by connecting the polar structure Pna2_1_ to the non-polar Pnna. As stated in^9^, the transition path connecting both structures will not have an effect in the final result. To this end, we choose a linear path from Pna2_1_ to Pnna through the following equations:

1 $$\begin{array}{c}{x}_{i}(\lambda )=\lambda {x}_{i}(Pna{2}_{1})+\mathrm{(1}-\lambda ){x}_{i}(Pnna)\\ {y}_{i}(\lambda )=\lambda {y}_{i}(Pna{2}_{1})+\mathrm{(1}-\lambda ){y}_{i}(Pnna)\\ {z}_{i}(\lambda )=\lambda {z}_{i}(Pna{2}_{1})+\mathrm{(1}-\lambda ){z}_{i}(Pnna)\end{array}$$

where *λ* goes from 0 to 1. The evolution of the electric polarization we obtained is depicted in Fig. 4(a). As we can observe, the electric polarization has a linear variation with the path connecting both structures (Polar - Non-polar), and the electric polarization obtained for the material is ≈29 *μC*/*cm*^2^, which is in good agreement with the experimental result of 15 *μC*/*cm*^2^ at 160 K^6^, and with the theoretical value of 25 *μC*/*cm*^2^ for GaFeO_3_^9^, indicating that AFO is a ferroelectric material.

In Fig. 4(b) we present the total energy difference relative to the Pna2_1_ symmetry, in the transition path from the polar Pna2_1_ to the non-polar Pnna. This information gives an estimative of the energy barrier for the system transit to the polar to the non-polar configuration. The Pna2_1_ is around 0.64 eV/f.u. lower in energy than the non-polar Pnna system, showing that the system lowers its total energy, distorting its centrosymmetric configuration, generating in this way an electric polarization. The presence of those energy barriers as pointed out by Stoeffler in ref.^9^, will lead to the presence of a hysteresis loop under an applied electric field. This behavior has also experimentally been observed for the AlFeO_3_ in a previous work by Santos *et al*. in ref.^5^.

The AFO crystal structure at 160 K is almost identical to that obtained at room temperature by Bouree *et al*.^4^. In fact, they have performed the measurements at 30 K and at room temperature and found that the crystal structure did not change the symmetry, maintaining the orthorhombic structure in the Pna2_1_ space group. In this sense, our calculated and measured polarizations are very similar to each other. The ferroelectric behavior of AFO was also demonstrated by pyroelectric, ferroelectric and PFM measurements^5,6,8^. In other words, this kind of bond interaction, mediated by the superexchange interaction between the Fe sites, creates weaker and stronger covalent bonds and moves the ions from their centrosymmetric positions, leading to the appearance of electric polarization^13,20^.

Using the one-dimensional electron density between oxygen ions and the cationic sites, depicted in Fig. 3(a–h) (down panel) and summarized in Table 2, we show the values of the distance and the minimum electron density (MED) for all cation-anion bonds. MED was used to parameterize the strength of the bonds between the neighbouring ions. It is possible to see that these chemical bonds are anisotropic, resulting in the configurations of shorter/longer and stronger/weaker bonds. The Fe1-O5, Fe2-O3, Al1-O6 and Al2-O1 bonds are the strongest bonds at the Fe1, Fe2, Al1 and Al2 sites, respectively. Additionally, it can be observed that these strongest bonds have a more covalent character, i.e., there is a significant overlap between the Fe(3*d*)/O(2*p*) orbitals. In this scenario, it is also possible to conclude that the main contribution to the net electric polarization is due to the Fe sites, whereas the Al1 and Al2 sites make weaker or no contribution to the net electric polarization; this is because Al does not have any valence electrons to contribute to the electric polarization, and the core electrons do not contribute to the electron polarization. The charge density observed around the Al1 and Al2 sites in the electron density maps (Fig. 3(c) and (d)) is almost completely due to the Fe^+3^ ions located at each site.

**Figure 5:**
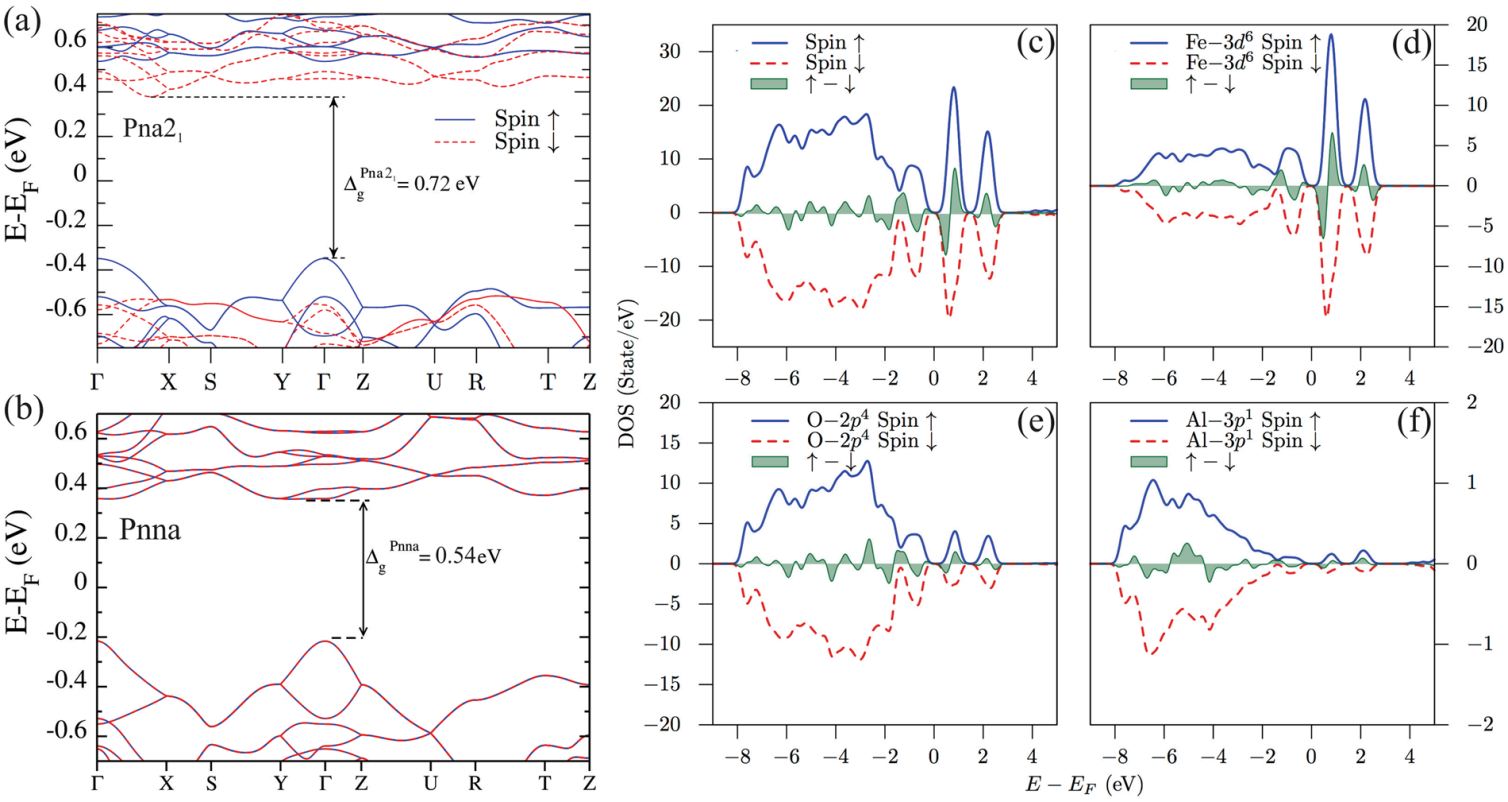
Band structure of bulk AlFeO_3_ along the principal high-symmetry directions in the Brillouin zone for (**a**) Pna2_1_ symmetry and (**b**) Pnna symmetry. The Fermi energy (E_*F*_) is set to zero. (**c**) Total density of states (DOS) of the bulk AlFeO_3_ crystal and projected density of states on the atoms (**c**) Fe, (**d**) O and (**e**) Al. In each figure, we present the up and down component and its difference. The Fermi level was set to zero. The total and projected density of states are only shown for the Pna2_1_ system.

To fully characterize the electronic properties of the systems, the band structure of the Pna2_1_ AFO composition was calculated along the high-symmetry lines, as depicted in Fig. 5(a). Initially, we conducted the calculations using an antiferromagnetic configuration. However, it is possible to observe a split in the spin polarization of the band structure, as presented in Fig. 5(a), with the solid blue lines for the majority spin and red dashed lines for the minority spins. Indeed, the system relaxes to a weak ferromagnetic configuration, in a similar way as that obtained for GaFeO_3_^9^. The overall band gap is close to 0.72 *eV* and is indirect with the top of the valence band located at the Γ point, and the bottom of the conduction band located in a valley between Γ and *X*, exhibiting opposite spin components. One important point is that this lift of the spin degeneracy of the band structure is due to the presence of the spontaneous polarization in the material. Using the same idea as we have already used to calculate the polarization of the system, we have calculated the band structure of the non-polar Pnna structure, as depicted in Fig. 5(b). As we see, the band structure of the Pnna symmetry is spin degenerated, and this system presents an indirect band gap around ≈0.54 *eV*.

The projected density of states (PDOS) analysis, presented in Fig. 5(c–f) only for the Pna2_1_, shows that the valence band is mainly derived from the overlap between the O(2*p*) and the Fe(3*d*) orbitals, that is also close to the results obtained for GaFeO_3_^9^. The s orbitals of Al^3+^, Fe*p* and O^2−^ and the Al(3*p*) has a small contribution to the valence band. The bottom of the conduction band arises predominantly from the Fe(3*d*) orbitals. Even considering a structure without any defects and no mixing between Fe*d*/Al*p* ions sites in the simulation, the up and down spin contributions are asymmetric, resulting in a net magnetization, implying that a weak ferromagnetic (or ferrimagnetic) state is induced. The net magnetic moments obtained for the Fe1 and Fe2 sites are 3.53 and 3.59 *μ*_*B*_, respectively. These values are similar to those reported by Saha *et al*.^21^ and Stoeffler^9^. The MEM and DFT calculations showed that the AFO composition has magnetic and ferroelectric polarizations, with both of these ferroic properties arising from the same ionic states, Fe^+3^(3*d*) and O^−2^(2*p*). This means the AFO should exhibit magnetoelectric coupling, as has already been addressed by experimental studies.

## Discussion

In summary, using the maximum entropy method applied to the X-ray diffraction data and *ab initio* density functional theory calculations, the microscopic origin of ferroic and multiferroic properties in the lead-free AlFeO_3_ compound was studied. Examining the obtained electron density distributions, we conclude that the chemical bonds between the Fe(3*d*) and O(2*p*) ions are anisotropic, leading to configurations of shorter/longer and stronger/weaker bonds and generating an electric polarization in AlFeO_3_. In fact, it is well-known that the displacement of the cations in the octahedral and tetrahedral environments can generate an electric polarization. Density of states calculations showed a magnetic polarization as a result of a weak ferromagnetic (or ferrimagnetic) ordering, even for the AlFeO_3_ with an antiferromagnetic structure. Thus, a resultant net magnetization could be observed. Band structure calculations for AlFeO_3_ found a gap of a 0.72 *eV*. This helps to explain the high conductivity behavior found in previous dielectric measurements. In this sense, even without the inclusion of disorder or defects in the calculations, together with the experimental data, our theoretical results show that the AlFeO_3_ compound is a multiferroic material and can exhibit a magnetoelectric coupling, as already addressed by experimental studies.

## Methods

### Experimental Methods

The AFO composition was synthesized by the high-energy ball milling route followed by a high-temperature solid state reaction (1450° C per 6 hour in the O2 atmosphere), as reported in previous works^5,6^. The sample was characterized by X-ray powder diffraction (XRPD) using a Shimadzu XRD-7000 diffractometer (Cu k *α* radiation). Crystallographic parameters and structure factors were refined in a Rietveld analysis using the RIETAN-FP software^22^. Electron density distributions were calculated by MEM using the structure factors obtained by Rietveld analysis. These calculations were performed using the Dysonomia^23^ software by means of a high-resolution grid (128 × 128 × 128 pixels) and using the limited-memory Broyden-Fletcher-Goldfarb-Shanno (L-BFGS) optimization algorithm^24^.

### Theoretical Calculations

*Ab initio* calculations were carried out using density functional theory (DFT), as implemented in the SIESTA code^25^. To represent the exchange-correlation energy, we used the generalized gradient approximation (GGA) of Perdew, Burke, and Ernzerhof (PBE)^26^. To accurately describe the density on the grid, we used 600 Ry for the mesh-cutoff, and the integrations over the Brillouin zone of the orthorhombic crystal were performed using a 6 × 6 × 6 k-point mesh. These parameters were sufficient for obtaining a well-converged total energy and all forces were optimized until their magnitudes were smaller than 0.04 *eV*/Å. The structures used in the calculations were obtained from the structural factors in the Rietveld analysis. To plot the crystal structure, electron density maps and one-dimensional electron density distributions, we used the VESTA software^27^.

The electric polarization was calculated using the modern theory of polarization together with the Berry phase method^28^, as implemented in Siesta code^29^, which can be performed to quantitatively estimate the polarization for a ferroelectric system in a given crystal phase. In our calculations, we used a discretized version of the polarization given by

2 $${P}_{e,||}=\frac{ife}{8{\pi }^{3}}{\int }_{A}d{k}_{\perp }\sum _{n=1}^{M}{\int }_{0}^{|{G}_{||}|}\langle {u}_{kn}|\frac{\partial }{\partial {k}_{||}}|{u}_{kn}\rangle d{k}_{||}$$

where *f* is the occupation, *e* is the electron charge, *M* is the number of occupied bands, and *u*_*kn*_ are the periodic Bloch functions. *G*_||_ is the shortest reciprocal vector along the chosen direction. A *k* – *point* sampling of 9 × 9 × 9 was used for the Berry phase calculation.
